# Histone acetylation and arachidonic acid cytotoxicity in HepG2 cells overexpressing CYP2E1

**DOI:** 10.1007/s00210-013-0942-4

**Published:** 2013-11-28

**Authors:** A. Holownia, R. M. Mroz, P. Wielgat, P. Jakubow, J. Jablonski, J. Sulek, J. J. Braszko

**Affiliations:** 1 Department of Clinical Pharmacology, Medical University of Bialystok, Waszyngtona 15A, 15-274 Bialystok, Poland; 2 Department of Pneumology, Medical University of Bialystok, Zurawia 14, 15-540 Bialystok, Poland; 3 Department of Toxicology, Medical University of Bialystok, Mickiewicza 2c, 15-089 Bialystok, Poland

**Keywords:** Arachidonic acid, CYP2E1, Cytotoxicity, Ethanol, HepG2 cells, Histone acetylation

## Abstract

The aim of this work was to assess the role of ethanol-derived acetate and acetate-mediated histone acetylation in arachidonic acid-induced stress in HepG2 cells and cells overexpressing CYP2E1. Cells were grown for 7 days with 1 mM sodium acetate or 100 mM ethanol; their acetylated histone proteins and histone deacetylase 2 expression was quantified using Western blot. Ethanol- or acetate-pretreated cells were also treated for 24 h with 60 μM arachidonic acid to induce oxidative stress. Cytotoxicity was estimated by lactate dehydrogenase release, 3-[4,5-dimethylthiazolyl-2] 2,5-diphenyltetrazolium bromide test, and by DNA damage, while oxidative stress was quantified using dichlorofluorescein diacetate. Cells grown with ethanol or acetate had increased acetylated histone H3 levels in both cell types and elevated acetylated histone H4 levels in cells overexpressing CYP2E1 but not in naïve cells. In cells overexpressing CYP2E1 grown with ethanol, expression of histone deacetylase 2 was reduced by about 40 %. Arachidonic acid altered cell proliferation and was cytotoxic mostly to cells engineered to overexpress CYP2E1 but both effects were significantly lower in cells pretreated with ethanol or acetate. Cytotoxicity was also significantly decreased by 4-methylpyrazole—a CYP2E1 inhibitor and by trichostatin—an inhibitor of histone deacetylases. In cells pretreated with acetate or ethanol, the oxidative stress induced by arachidonic acid was also significantly lower. Our data indicate that histone hyperacetylation may in some extent protect the cells against oxidative stress. It is possible that acetate may act as an antioxidant at histone level. This mechanism may be relevant to alcohol-induced liver injury.

## Introduction

Ethanol is metabolized in the liver by alcohol dehydrogenase, cytochrome P4502E1, and catalase-producing acetaldehyde, which is further oxidized by aldehyde dehydrogenase to acetate. Acetate is considered as not toxic and it is mostly removed in Krebs cycle as acetylCoA (Cederbaum 2012); however, it was shown that ethanol-derived acetate can acetylate different proteins, and acetylated cytosolic, mitochondrial, and nuclear hepatic proteins have been detected in liver samples from patients with alcoholism and in experimental animals intoxicated with ethanol (Shepard and Tuma 2009). Ethanol-derived acetate can acetylate such important proteins like tumor suppressor p53 protein, sterol response element binding protein-1c, peroxisome proliferator-activated receptor γ coactivator α (PGC-1α), α-tubulin, mitochondrial acetyl CoA synthetase 2, and others (Shepard and Tuma 2009). The first hyperacetylated liver protein which was detected in an animal model of both acute and chronic alcohol intoxication was a nuclear histone H3 protein (Park et al. 2003). Histones are important in all aspects of cell physiology and pathology because they are involved in regulation of gene transcription. Histone–DNA complexes are usually transcriptionally activated by histone acetylation, but functional regulation of histone protein is also controlled by methylation and other reactions important in epigenetic regulations (Zentner and Henikoff 2013). Their role in alcohol-induced liver injury is still not assessed but several evidences stress important role of histone acetylation in inflammatory signaling and it seems that histone acetylation may also be relevant to alcoholic hepatitis and cirrhosis. It was shown that acetate generated in alcohol metabolism increased histone acetylation and expression of inflammatory cytokines pointing to important role of hyperacetylation of core histones in alcoholic hepatitis (Mandrekar and Szabo 2009; Kendrick et al. 2010; Mandrekar 2011). Histones can be transcriptionally silenced by deacetylation, which is mediated by histone deacetylases (HDACs) and it was shown that deregulation of HDAC, probably due to oxidative stress, could play a major role in the binge alcohol-induced hepatic steatosis and liver injury (Kirpich et al. 2012). Post-alcoholic liver damage is also associated to oxidative stress mediated in major part by CYP2E1 (Albano 2006). The role of histones in oxidative stress in less known, although recent data indicate that HDAC inhibition and histone hyperacetylation may significantly suppress oxidative stress induced by β-hydroxybutyrate (Shimazu et al. 2013). The objective of this work was to alter histone acetylation in cultured naïve HepG2 cells and HepG2 cells overexpressing alcohol metabolizing enzyme—CYP2E1, by growing the cells in medium supplemented with ethanol or with acetate and to compare the effect of oxidative stress induced by arachidonic acid (AA) on cell growth and survival.

## Materials and methods

### Cells and reagents

The human hepatoblastoma cell subline-HepG2 stably transfected with human CYP2E1 and HepG2 cells transfected with an empty vector with undetectable CYP2E1 activity were kindly provided by Professor A. Cederbaum of the Department of Pharmacology and Biological Chemistry, Mount Sinai School of Medicine in New York, USA. Cells were grown in minimal essential medium (MEM) supplemented with 10 % fetal bovine serum, penicillin (100 U per ml), and streptomycin (100 μg per ml), at 37 °C in a humidified atmosphere of 5 % CO_2_. For maintaining CYP2E1 expression, the medium was additionally supplemented with G418-sulfate (200 μg per ml), the antibiotic used for the selection process.

### Cell treatment

Cells were grown for 1, 3, or 7 days in medium supplemented with 1 mM sodium acetate (Sigma Chemical Co) or with 100 mM ethanol and media were refreshed every 24 h. Decline in ethanol levels after 24 h incubation was quantified using gas chromatography with mass spectrometry detection (Tsukamoto et al. 1998), and 24 h after ethanol treatment, ethanol levels in the culture media were decreased by about 65 %. Both HepG2 cells and transduced cells produced significant amounts of acetaldehyde and acetate as detected by gas chromatography–mass spectrometry, which confirmed earlier observations (Marselos et al. 1987; Wu et al. 2006). In some experiments, 5 mM 4-methylpyrazole (4MP) was used for 24 h to inhibit CYP2E1 and 100 ng/ml trichostatin (TSA; 24 h) to inhibit HDACs. None of the above treatments resulted in significant cytotoxicity basing on culture medium lactate dehydrogenase activity (LDH) assay. After preincubation of cells with ethanol or with acetate, cells were seeded into 6- or 25-well plates and were grown for additional 24 h in MEM medium supplemented with 60 μM AA (Sigma Chemical Co).

### Subcellular fractions and histone isolation

To isolate cytosolic and nuclear fractions, cells were scraped, centrifuged, resuspended in cold hypotonic buffer containing 10 mM HEPES, pH 7.9, 1.5 mM MgCl_2_, 10 mM KCl, 50 mM dithiothreitol, 100 mM phenanthroline, 1 mg/ml pepstatin, 100 mM trans-epoxysuccinyl-L-leucylamido-(4-guanidino)butane, 100 mM 3,4-dichloroisocoumarin, 10 mM NaF, 100 mM sodium orthovanadate and 25 mM β-glycerophosphate, and centrifuged at 14,000×*g* for 5 min at 4 °C. Then, cells were lysed in a solution of the same buffer containing 0.2 % (*v*/*v*) Nonidet P-40 for 10 min on ice and subsequently centrifuged at 14,000×*g* for 10 min at 4 °C. The supernatant was collected as cytosolic extract. The remaining pellet was resuspended in extraction buffer (20 mM HEPES, pH 7.9, 420 mM NaCl, 1.5 mM MgCl_2_, 0.2 mM EDTA, 25 % (*v*/*v*) glycerol, 100 mM 3,4-dichloroisocoumarin), incubated for 15 min at 4 °C, and centrifuged at 14,000×*g* for 10 min at 4 °C. The supernatant including soluble nuclear protein was collected as nuclear extract.

Acid extraction of histones was performed in cells treated for 30 min in ice with lysis buffer 10 mM HEPES, pH 7.9, 1.5 mM MgCl_2_, 10 mM KCl, 0.5 mM DTT, and 1.5 mM phenylmethylsulfonyl fluoride and hydrochloric acid at a final concentration of 0.2 M, and subsequently, lysed cells were centrifuged at 11,000×*g* for 10 min at 4 °C. Supernatant containing acid-soluble proteins was dialyzed for 1 h, against 0.1 M acetic acid and then overnight against H_2_O and frozen until assayed (Chadee et al. 1999). Quantification of acetylated histones with broad range anti-acetylated histone antibodies and Western immunoblotting indicated that the highest increase in acetylated histones was obtained after 7 days of cell treatment with ethanol or acetate, thus 7 days treatment was used in all further experiments.

### Western immunoblotting

Specific proteins were analyzed by sodium dodecyl sulfate-polyacrylamide gel electrophoresis/immunoblotting with appropriate rabbit antibodies (Santa Cruz Biotechnology, Heidelberg, Germany) recognizing HDAC2 protein (cytosol; sc-81599 antibody) or acetylated (Ser 11 and/or Lys 115) histone H3 (AcH3; sc-33361 antibody) and acetylated (Ser 1, Lys 5, Lys 8 and Lys 12) histone H4 (AcH4; sc-34263 antibody) (acid-extracted samples). Proteins were separated along with specific protein standards and molecular weight markers (Bio-Rad, Warsaw, Poland) in 10 % (HDAC2) or 20 % (acetylated histones) polyacrylamide gels. Gels were transferred onto 0.45-μm PVDF membranes (BioRad, Warsaw, Poland). Species-specific horseradish peroxidase or alkaline phosphatase secondary antibodies were purchased from Santa Cruz or Sigma, respectively. Changes in AcH3 and AcH4 histone levels were analyzed comparing to total histone H3 and histone H4 levels (total human histone H3 and H4 antibodies were from Santa Cruz Biotechnology, Heidelberg, Germany; sc-8654 antibody and sc-10810 antibody, respectively). Loading controls for HDAC were done using ß-actin. Protein bands were quantified using Quantity One software (BioRad, Warsaw, Poland). Samples were run in duplicate on each gel and mean values were expressed as 100 relative units ± SD to compare data from different experiments.

### Determination of cell viability

Cell viability was primarily tested by time-dependent LDH release to the culture medium using LDH cytotoxicity kit (ScienCell, Carlsbad, USA), and then also by 3-[4,5-dimethylthiazolyl-2] 2,5-diphenyltetrazolium bromide (MTT) assay (Niks and Otto 1990) and by flow cytometry estimation of cell growth and cytotoxicity (Shapiro 1988). For the experiments, control cells or cells pretreated with ethanol or acetate were seeded on 24-well plates and treated with AA and LDH activity in the culture media was quantified after 1, 3, 6, 12, and 24 h according to manufacturers' instruction. LDH data were compared to total enzyme activity obtained after cell sonication.

MTT assay and flow cytometry were performed after 24 h of cell treatment with AA. In MTT test, changes in absorbance due to formazone production in viable cells were measured after 24 h of AA treatment at 570 nm, with 630 nm as a reference wavelength. Cell viability was estimated as a percentage of the control. To quantify alterations in cell growth and cytotoxicity by flow cytometry, cells were stained for 30 min with propidium iodide (PI; 50 μg per ml) in TRIS buffer (100 mM; pH 7.5), containing 0.1 % potassium cyanide, 0.01 % NP-40, 40 μg per ml Type III-A RNAse, and 0.1 % NaN_3_. DNA profiles in particular cells and cell cycle analysis were performed on an aligned Coulter Epics Profile Flow Cytometer (Coulter, Hialeah, FL, USA) equipped with an argon laser operating at 488 nm with adjusted forward angle- and side light-scatter. PI fluorescence was measured in 5,000–10,000 cells and DNA fluorescence histograms were analyzed by cells cycle software (MultiCycle, Phoenix Flow Systems Inc, San Diego, CA, USA). The cells were quantified by their relative distribution in the hypodiploid (“early” GO/G1 zone of the DNA fluorescence histograms), diploid (G0/G1 zone—pre-DNA synthesis/resting), S-phase (DNA synthesis), and G2/M (post DNA synthesis/mitosis) phases of the cell cycle. The percentage of cells in the subdiploid regions of histograms was considered as an index of cytotoxicity while S + G2/M cell numbers were quantified as proliferation index.

### Oxidative stress

Intracellular generation of reactive oxygen intermediates was quantified in control cells and in cells treated with AA for 1, 3, 6, 12, and 24 h using dichlorodihydrofluorescein diacetate (H_2_DCFDA; Sigma Chem. Co.) (Ubezio and Tivoli 1994). Cells were loaded with 5 μM H_2_DCFDA for 30 min, washed, resuspended in phosphate-buffered saline, and assayed by flow cytometry. Green dichlorofluorescein (DCF) fluorescence was captured on Fl1 and registered as histograms of fluorescence distribution.

### Protein levels

Homogenate proteins were measured using BCA kit (Sigma-Aldrich, Poznan, Poland).

### Data analysis

Statistical analysis was performed with a statistics package—Statistica 6.0 software (Statsoft; Cracow, Poland) using one-way or two-way ANOVA followed by Bonferroni post tests for selected pairs of data. Results were expressed as mean of 6 to 10 assays ± S.D. *P* values less than 0.05 were considered statistically significant.

## Results

**Fig. 1:**
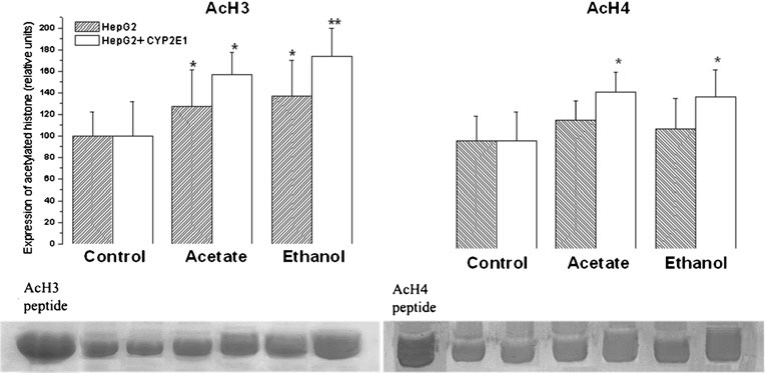
Acetylated histone H3 (*AcH3*) and histone H4 (*AcH4*) in HepG2 cells and HepG2 cells overexpressing CYP2E1 grown for 1 week in culture medium supplemented with 1 mM acetate or 100 mM ethanol. Figures represent mean results ± SD. Data are relative values (digitized relative density) expressed as percentages over untreated cells taken as 100 %. Representative Western blot pictures are included. Statistically significant differences from controls are indicated as: *P < 0.05 or **P < 0.01

Figure 1 shows acetylated histone H3 (AcH3) and acetylated histone H4 (AcH4) in HepG2 cells and HepG2 cells overexpressing CYP2E1 grown for 7 days in culture medium supplemented with 1 mM acetate or 100 mM ethanol. Both treatments increased AcH3 in transduced and nontransduced cells, while AcH4 levels were significantly increased in cells overexpressing CYP2E1 grown with acetate or ethanol, but not in naïve HepG2 cells. The highest expression of acetylated histones (increase by 73 %; *P* < 0.01) was detected in H3 protein in cells overexpressing CYP2E1 grown with ethanol and slightly lower AcH3 levels (increase by 53 %; *P* < 0.05) were found in the same cell type grown with acetate. Lower but statistically significant increases were observed in AcH4 levels, in cells engineered to overexpress CYP2E1 treated with ethanol or acetate (increase by about 40 %; *P* < 0.05 in both groups), while in nontransduced cells AcH4 levels were not affected.

**Fig. 2:**
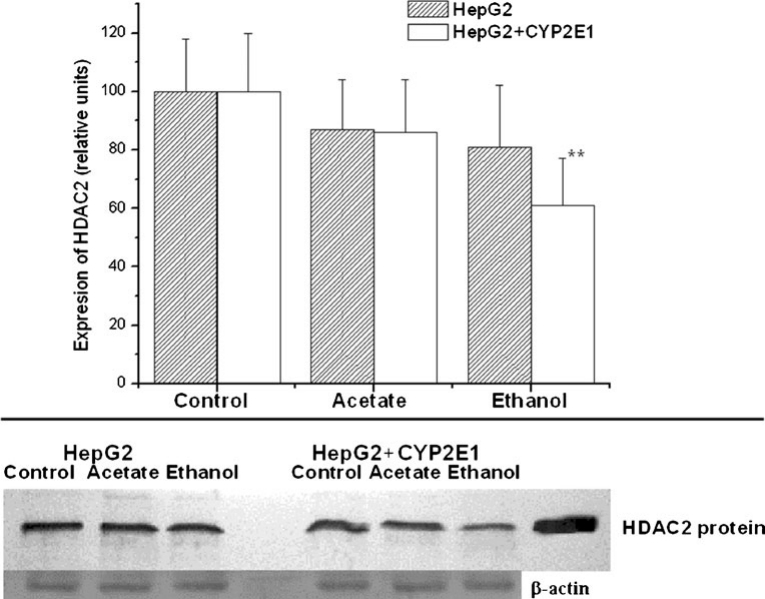
Histone deacetylase 2 (HDAC2) expression in HepG2 cells and HepG2 cells overexpressing CYP2E1 grown for 1 week in culture medium supplemented with 1 mM acetate or 100 mM ethanol. Figures represent mean results ± SD of five experiments. Data are shown as relative values (digitized relative density) expressed as percentages over untreated cells taken as 100 %. Representative Western blot picture is also included. Statistically significant difference from control is indicated as: **P < 0.01

HDAC expression was reduced by about 40 % (*P* < 0.01) only in cells overexpressing CYP2E1 treated with ethanol (Fig. 2).

**Fig. 3:**
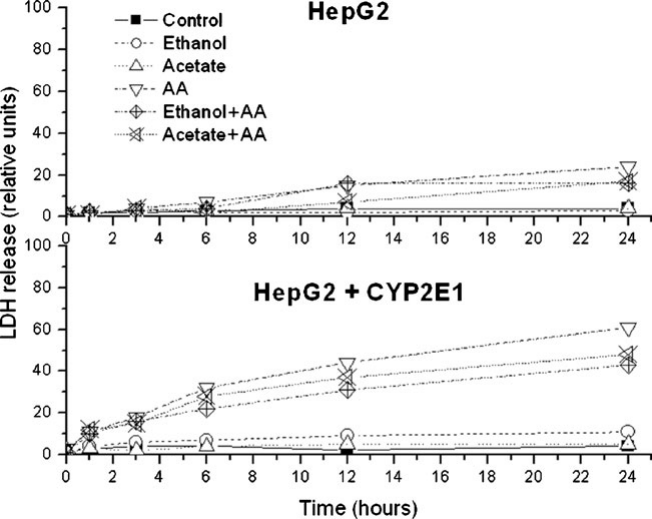
The effect of arachidonic acid (*AA*; 60 μM) on viability of HepG2 and HepG2 cells overexpressing CYP2E1. Cell viability was assessed 1, 3, 6, 12, and 24 h after AA treatment using lactate dehydrogenase (LDH) assay. The same AA treatment was also done in cells grown prior to AA treatment for 1 week in culture medium supplemented with 1 mM acetate (*Acetate + AA*) or 100 mM ethanol (*Ethanol + AA*). Values of maximal LDH release reflecting total cell destruction were obtained after cell sonication. Each plotted point is a mean value from six experiments. Cytotoxicity data after 24 h of AA treatment are shown also in Table 1

Figure 3 shows time-dependent LDH release from HepG2 cells and HepG2 cells overexpressing CYP2E1 treated with AA and also from both cell types grown for 1 week prior to AA treatment with 100 mM ethanol or 1 mM acetate. LDH release after 24 h of AA treatment is also shown in Table 1.

**Table 1:** Arachidonic acid (AA; 60 μM, 24 h)-induced lactate dehydrogenase (LDH) release, cytotoxicity, alterations in cell proliferation (S + G2/M cells), and oxidative stress (DCF) in HepG2 cells and HepG2 cells overexpressing CYP2E1 and in the same cell types grown for 1 week prior to AA treatment in the culture medium supplemented with 100 mM ethanol or 1 mM acetate. In some experiments, cells were additionally treated for 24 h with both AA and with CYP2E1 inhibitor, 4-methylpyrazole (4MP; 5 mM) or pretreated for 24 h before AA with trichostatin (TSA, 100 ng/ml) to inhibit histone deacetylases. LDH activity in the culture medium was measured using a LDH cytotoxicity kit and results are expressed as a fraction of total enzyme activity acquired after cell sonication. To quantify oxidative stress, the cells were stained with dichlorofluorescein diacetate (DCF) and green fluorescence was captured in viable cells only using flow cytometry. Alterations in cell growth and AA cytotoxicity were tested in flow cytometry after cell staining with propidium iodide (PI) and analysis of DNA-PI fluorescence histograms using multicycle software. Table shows fractions of damaged cells (subdiploid—GO/G1zone of the DNA fluorescence histograms; Fig. 7—“early G0/G1 cells”) and percentages of proliferating cells in S(DNA synthesis) + G2/M (post-DNA-synthesis/mitosis) phases of cell cycle (Fig. 7). Each PI fluorescence distribution histogram was derived from analysis of at least 5,000 cells and six samples were analyzed in each group. Table shows mean values ± SD

	HepG2				HepG2 + CYP2E1			
	LDH	Damaged cells (%)	Proliferation (S + G2/M cells; %)	DCF	LDH	Damaged cells (%)	Proliferation (S + G2/M cells; %)	DCF
Control	5 ± 3	5 ± 1	60 ± 8	100 ± 18	7 ± 2	5 ± 2	65 ± 11	134 ± 21
Ethanol	8 ± 2	4 ± 1	62 ± 9	108 ± 15	9 ± 4	5 ± 2	66 ± 10	158 ± 33
Acetate	6 ± 2	6 ± 1	61 ± 9	106 ± 12	8 ± 4	7 ± 1	62 ± 13	130 ± 26
4MP	11 ± 6	7 ± 2	48 ± 9^*^	111 ± 16	13 ± 8	13 ± 4^*^	47 ± 11^**^	137 ± 23
TSA AA	10 ± 7 20 ± 8^**^	9 ± 2 11 ± 2^**^	45 ± 9^*^ 45 ± 11	104 ± 15 121 ± 19	12 ± 7 64 ± 11^**^	11 ± 7^*^ 35 ± 5^**^	46 ± 14^*^ 30 ± 18^**^	130 ± 32 571 ± 72^**^
AA + 4MP	21 ± 9^**^	12 ± 4^**^	41 ± 10^*^	126 ± 21	43 ± 12^** †<††^	22 ± 5^**†<^	33 ± 13^**^	243 ± 39^**††<^
AA + TSA Ethanol + AA	22 ± 12^**^ 20 ± 5^**##^	13 ± 6^**^ 9 ± 2^**##^	42 ± 14^*^ 44 ± 14	112 ± 23 124 ± 19	47 ± 11^** †⊥^ 47 ± 10^**##††^	21 ± 11^** †^ 20 ± 6^**#††^	38 ±12^**†^ 34 ± 17^**^	355 ± 51^**††⊥⊥^ 426 ± 65^**##††^
Acetate + AA	21 ± 7^**++^	11 ± 24^**++^	65 ± 12^†^	122 ± 20	51 ± 9^**#++††^	18 ± 4^**+††^	50 ± 17	462 ± 78^**##++††^

*P < 0.05 (vs. corresponding control); **P < 0.01 (vs. corresponding control); ^#^ *P* < 0.05 (vs. corresponding data from ethanol-treated cell); ^##^ *P* < 0.01 (vs. corresponding data from ethanol-treated cells); ^+^ *P* < 0.05 (vs. corresponding data from acetate-treated cells); ^++^ *P* < 0.01 (vs. corresponding data from acetate-treated cells); ^†^ *P* < 0.05 (vs. corresponding data from AA-treated cells);^††^ *P* < 0.01 (vs. corresponding data from AA-treated cells); ^<^ *P* < 0.05 (vs. corresponding data from 4MP-treated cells);^<<^ *P* < 0.01 (vs. corresponding data from 4MP-treated cells); ^⊥^ *P* < 0.05 (vs. corresponding data from TSA-treated cells); ^⊥⊥^ *P* < 0.01 (vs. corresponding data from TSA-treated cells)

Neither ethanol nor acetate released LDH in both types of cells. AA treatment increased (*P* < 0.01) LDH release to the culture medium of HepG2 cells treated with AA for 24 h (Fig. 3, Table 1) but in cells overexpressing CYP2E1 cell damage was increased already after 6 h of AA treatment and was still rising with time reaching at 24 h of AA treatment values about three times higher than in naïve HepG2 cells treated with AA (*P* < 0.01) or in control cells (*P* < 0.01). 4MP was without effect on LDH release induced by AA in HepG2 cells, while in transduced cells, AA cytotoxicity was decreased (Table 1) when AA was applied together with 4MP (*P* < 0.05) or when the cells were pretreated with TSA (*P* < 0.05). Cells overexpressing CYP2E1 but not naïve cells grown with acetate or ethanol prior to AA treatment were also more resistant to AA (*P* < 0.01 and *P* < 0.01 for ethanol and acetate-pretreated cells, respectively).

**Fig. 4:**
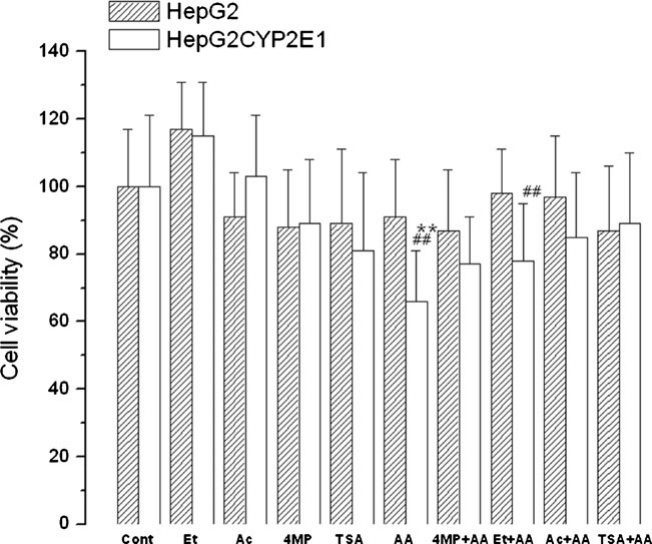
The effect of arachidonic acid (*AA*; 60 μM; 24 h) on viability of HepG2 and HepG2 cells overexpressing CYP2E1. Cell viability was assessed with MTT test. The same treatment was done in cells grown for 1 week with medium supplemented with 1 mM acetate or 100 mM ethanol. Results from six independent experiments are expressed as mean percentages of viable cells ± SD and control was taken as 100 %. Statistically significant difference from the control is indicated as *P < 0.05 and ^##^ *P* < 0.01—from ethanol-treated cells

Cytotoxicity of AA applied for 24 h to the cells was also tested by MTT test (Fig. 4). In HepG2 cells overexpressing CYP2E1, AA significantly decreased cell viability (by about 37 %; *P* < 0.01). This effect was observed neither in naïve HepG2 cells nor in CYP2E1 overexpressing cells grown for 1 week with ethanol or acetate. Cytotoxicity of AA in transduced cells was abolished not only by CYP2E1 inhibitor-4MP, but also by cell pretreatment with acetate or HDAC inhibitor—TSA (Table 1). In cells grown with ethanol prior to AA treatment, cytotoxicity of AA was slightly lower than in naïve cells but still significant (*P* < 0.01) comparing to ethanol-only treated cells.

**Fig. 5:**
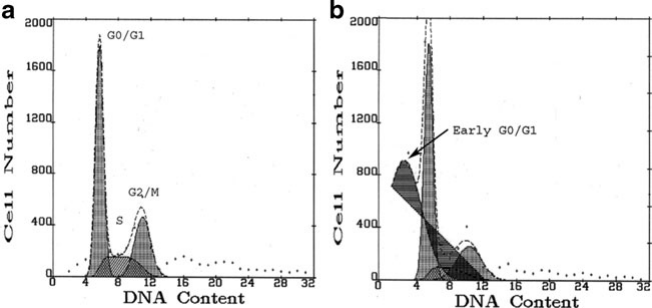
Representative flow cytometry histograms of propidium iodide fluorescence distributions (MultiCycle transformation) in HepG2 cells overexpressing CYP2E1 (**a**) and in the same cells treated for 24 h with arachidonic acid (**b**). The cells were quantified by their relative distribution in the damaged-subdiploid GO/G1 zone of the DNA fluorescence histograms (early G0/G1 cells), diploid (G0/G1 zone)—pre-DNA synthesis/resting, S-phase—DNA synthesis, and G2/M—post-DNA-synthesis/mitosis phases. Each histogram was derived from analysis of 5,000 cells and six samples were analyzed in each group. Quantification of cytotoxicity (early G0/G1 cells) and proliferation (S + G2/M cells) is shown in Table 1

Table 1 shows flow cytometry data of PI fluorescence reflecting cytotoxicity and proliferation of HepG2 cells or HepG2 cells overexpressing CYP2E1, and the same cell types grown for 1 week with 100 mM ethanol or 1 mM acetate and then treated for 24 h with AA as described in Materials and methods. Damaged cells were assessed as “early” G0/G1 cells and cell proliferation was quantified as S + G2/M cells (Fig. 5). Twenty-four hours of cell treatment with AA resulted in cytotoxicity in both cell types, but significantly higher toxicity was again observed in cells overexpressing CYP2E1. In HepG2 cells, AA treatment produced about 11 % of dead cells while in cells overexpressing CYP2E1 approximately 35 % of cells were damaged. AA cytotoxicity was lower in CYP2E1-expressing cells, which prior to AA treatment had grown with ethanol (20 % of dead cells; *P* < 0.01) or with acetate (18 % of dead cells, *P* < 0.01), while in HepG2 cells, such protective effects were not observed. 4MP decreased AA cytotoxicity by about 35 % (*P* < 0.05) in transduced cells only and similar effect was observed when transduced cells were pretreated with TSA (*P* < 0.05).

AA decreased proliferating cell numbers by about 25 % (*P* < 0.05) and 54 % (*P* < 0.01) in HepG2 cells and HepG2 cells expressing CYP2E1, respectively, and this effect was not significant in cells grown in acetate-supplemented media. Both 4MP and TSA decreased cell proliferation by about 30 %.

**Fig. 6:**
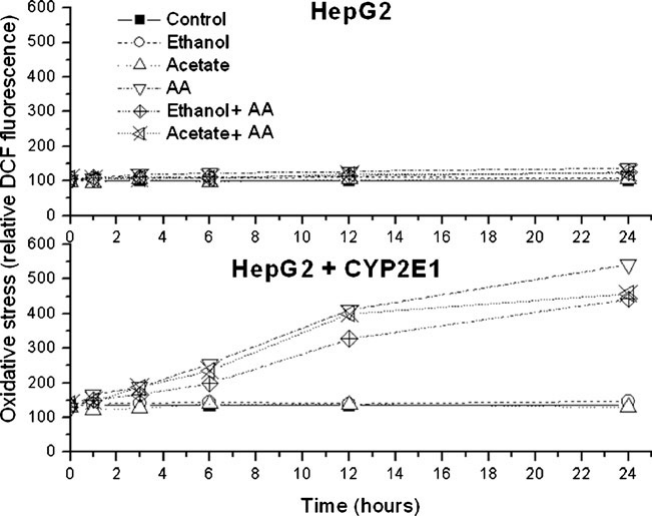
The effect of arachidonic acid (*AA*; 60 μM) on oxidative stress in HepG2 and HepG2 cells overexpressing CYP2E1. Oxidative stress was assessed 1, 3, 6, 12, and 24 h after AA treatment using flow cytometry detection of dichlorofluorescein diacetate fluorescence. The same AA treatment was done in cells grown prior to the treatment for 1 week in culture medium supplemented with 1 mM acetate (*Acetate + AA*) or 100 mM ethanol (*Ethanol + AA*). Mean DCF values after 24 h of AA treatments are also shown in Table 1

**Fig. 7:**
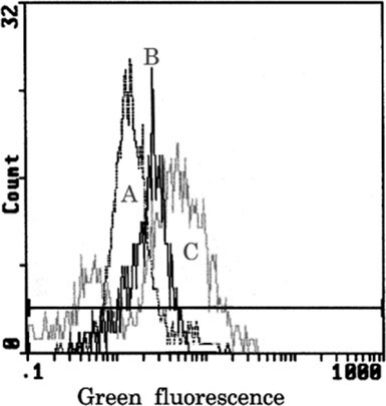
Histograms of DCF fluorescence reflecting oxidative stress in HepG2 cells overexpressing CYP2E1 (*A*) and the same cells treated for 6 (*B*) or 24 h (*C*) with 60 μM arachidonic acid (AA). After 6 h of cell treatment, increased oxidative stress (right shifted single peak fluorescence histogram B vs. A) was observed and after 24 h DCF fluorescence histograms were broad and double-peak representing (*left part* of histogram C) dead cell and (*right part* of histogram C) alive cells with high levels of oxidative stress

Ethanol itself only slightly but not significantly increased DCF fluorescence in nontransduced and transduced cells (Fig. 6, Table 1) while AA greatly increased oxidative stress only in cells overexpressing CYP2E1 reaching more than fourfold increase (*P* < 0.01) after 24 h of treatment. In transduced cells but not in HepG2 cells, DCF fluorescence was increased already after 3 h of AA treatment (Fig. 6). In contrast to naïve cells, in transduced cells treated with AA a double peak fluorescence histograms appeared (Fig. 7, histogram C) representing two cell subpopulations—dead cells in the left part of histogram C and alive cells on the right side of the histogram C. AA-induced oxidative stress in transduced cells was reduced by 4MP and slightly less decreased by cell pretreatment with TSA (Table 1) but the fluorescence was not normalized. In cells overexpressing CYP2E1 grown with ethanol or with acetate prior to AA treatment, oxidative stress induced by AA was lower than in naïve cells, respectively, by 25 % (*P* < 0.01) and by 19 % (*P* < 0.01).

## Discussion

Our data show that in cultured HepG2 cells grown for 7 days with ethanol or acetate, acetylated histone proteins are increased. Recent data point to alterations in histone acetylation as a transcriptional mechanism modulating negative effects of oxidative stress. It was shown that β-hydroxybutyrate increased histone acetylation and provided substantial protection against oxidative stress in experimental animals (Shimazu et al. 2013). Alterations in histone signaling were also reported in alcohol-induced liver injury (Kendrick et al. 2010; Mandrekar 2011; Seth et al. 2011), but the role of histone acetylation in alcohol-induced hepatitis or cirrhosis is less clear. Recent experimental evidences show that ethanol increases gene-selective association of acetylated H3-Lys 9 in the absence of global histone acetylation (Park et al. 2012). It was also shown that ethanol-induced acetylation of histone H3 in hepatocytes was related to oxidative stress (Choudhury et al. 2010). Liver cells are the main ethanol-metabolizing cells within the body; thus, ethanol generates oxidative stress mostly in hepatocytes. Ethanol-derived acetate can increase histone acetylation not only due to the elevated levels of ethanol-derived acetate but also by stimulation of histone acetyltransferases and inhibition of HDAC activity. HDACs are important regulators of many oxidative stress pathways including those involved with sensing and coordinating the cellular response to oxidative stress (Rahman et al. 2004). In our cells, decreased expression of relevant cytosolic HDAC isoenzyme HDAC2 was observed, in transduced cells treated with ethanol, although a tendency toward reduced HDAC protein levels could be observed also in cells grown with acetate. According to Agudelo et al. (2011), ethanol may increase HDAC2 expression in cultured neural cells, while in ischemia/reperfusion hepatocyte injury, activities of nuclear HDAC1 and HDAC4 are significantly decreased (Evankovich et al. 2010). It seems that lower HDAC2 expression in our cells and possibly also lower HDAC activity may play a role in histone hyperacetylation and may be due to oxidative stress associated with CYP2E1 expression. On the other hand, it was shown that HDAC inhibitors protect neuronal cells from oxidative stress-induced damages (Langley et al. 2008) and against in vitro cytotoxicity (Kang et al. 2005), and it appear that such mechanism may be extended to other diseases that share both oxidative stress and inflammation. Our data confirm that HDAC inhibitors may act as antioxidants, since TSA significantly reduced oxidative stress induced by AA.

AA is involved in oxidative stress and in inflammatory response in alcohol-induced liver injury (Mottaran et al. 2002). AA metabolism proceeds mostly via the CYP450 pathway and generates epoxyeicosatrienoic acids and monohydroxylated products (Caro and Cederbaum 2006). Cultured hepatoblastoma cells grown in medium supplemented with AA have altered inflammatory gene expression but die due to oxidative stress (Xu et al. 2003; Caro and Cederbaum 2006; Jimenez-Lopez et al. 2008). AA cytotoxicity can be definitely associated with the expression of ethanol metabolizing CYP450 isoenzyme—CYP2E1 (Caro and Cederbaum 2006; Jimenez-Lopez et al. 2008; Zhuge 2008), and our results confirm that AA cytotoxicity in HepG2 cells overexpressing CYP2E1 is mediated by oxidative stress, which is associated to CYP2E1 activity. However, when transduced cells were preincubated with ethanol or acetate, AA cytotoxicity was significantly lower or even abolished. Our data show that cells grown with ethanol or acetate have increases acetylated histone H3 while elevated acetylated histone H4 levels were detected only in transduced cells. Our data confirm earlier reports (Marselos et al. 1987; Wu et al. 2006) indicating that HepG2 cells metabolize ethanol to acetate, and show that that both extracellular acetate and intracellular acetate generated in ethanol metabolism may increase histone acetylation. On the other hand, an increase in acetylated histones which was observed in our cells was rather limited comparing to other published data, since according to (Choudhury et al. 2010) ethanol applied for a few hours to cultured hepatocytes increases their acetylated H3 levels by three- to eightfold depending on time span of alcohol exposure and on ethanol concentrations. In this study, maximal increase of acetylated H3 was observed after 24 h of cells treatment (Choudhury et al. 2010). We were not able to detect hyperacetylated H3 and H4 histones in our cells treated with acetate or ethanol for 24 h (results not shown), but it appears that apart from different methods of histone isolation and analysis, histone acetylation may vary significantly between cell types and should be examined in each experimental setting.

We focused on general indices of cell growth, proliferation, cytotoxicity, and oxidative stress. AA was cytotoxic to both cell types, but remarkably higher toxicity was observed in CYP2E1 expressing cells. Such effect was already described in similar cellular models (Wu and Cederbaum 2009) and our data confirm that at relatively high AA concentrations there is substantial oxidative stress leading to necrotic cell death. AA cytotoxicity to transduced cells was significant already after about 3 h of exposure. In cells that did not express CYP2E1, similar cytotoxicity was attained after 24 h of AA treatment. Inhibition of CYP2E1 by 4MP or TSA significantly reduced or even abolished AA cytotoxicity, depending on the assay, confirming the role of CYP2E1 and histones in AA cytotoxicity. It should be noticed that plots of AA-induced LDH release reflecting cytotoxicity and plots of DCF fluorescence reflecting intracellular oxidative stress are very similar, although they reflect two distinct processes. DCF assay shows intracellular prooxidative changes that take place in viable cells while LDH release is reflecting cell membrane damage leading to cell death. Similar plots may indicate that oxidative stress plays a major role in cell death. It was previously shown that AA toxicity (both apoptotic and necrotic) in HepG2 cells expressing CYP2E1 may be abolished by free radical spin traps (Pérez and Cederbaum 2001) or by overexpression of antioxidant superoxide dismutase (Pérez and Cederbaum 2003). Apart from direct cytotoxicity, AA can alter intracellular signaling. It was shown that AA markedly increases AP-1 binding to DNA in HepG2 cells and this effect was potentiated by oxidative stress (Bai and Cederbaum 2000). Exogenous AA has also been shown to alter p38 mitogen-activated protein kinase pathway in neural cells (Wood et al 2000) and to induce K(+) efflux via K^+^-Cl^−^-cotransport in HepG2 cells (Lee 2009). Alterations in intracellular ion balance and kinase signaling may be important to cell growth and survival; however, at higher AA concentrations, such effects appear to be less relevant due to critical role of oxidative stress, cell membrane damage, and rapid necrotic cell death.

In our experiments, AA cytotoxicity was significantly lower in transduced cells, which prior to AA treatment had grown with ethanol or acetate but relatively low or even no cytotoxicity at all was also noticed in cells pretreated with 4MP or TSA. These data stress the role of CYP2E1 in mechanisms of AA cytotoxicity, but also point to the influence of histones which seem to play important role in survival of cells subject to oxidative stress. TSA is able to rapidly increase acetylated histone levels in cultured liver-derived cells (Carlisi et al. 2008) and in longer treatment it can induce apoptosis (Buurman et al. 2012). It was also shown that histone hypearcetylation can increase antioxidant defense (Zelko et al. 2011). AA metabolites are also able to change histone H3 and in some extent also histone H4 acetylation profile (Doyle and Fitzpatrick 2010). On the other hand, cyclooxygenase 1 involved in AA metabolism and inflammation is transcriptionally regulated by histone acetylation (Taniura et al. 2002). It is possible that in cells treated with external AA signaling pathways related to inflammation are significantly affected, but this mechanism seems to play a role in initial phases preceding cell membrane damage and should be studied in viable cells using more sensitive methods. Another possibly important observation is that AA produced also substantial but significant toxicity also in nontransduced HepG2 cells and this effect was not altered by preincubation of cells with ethanol or acetate which suggest another mechanism, possibly not related to oxidative stress.

In conclusion, our data show that histone hyperacetylation can, in some extent, protect the cells against oxidative stress and AA cytotoxicity. It is established that ethanol can generate oxidative stress at CY2E1 level. Our data indicate that ethanol metabolite—acetate, can shift histone acetylation toward hyperacetylation to act as antioxidant at histone level. This mechanism may be relevant in alcohol-induced liver injury.
